# Flexural and Free Vibration Analysis of CNT-Reinforced Functionally Graded Plate

**DOI:** 10.3390/ma11122387

**Published:** 2018-11-27

**Authors:** Md Irfan Ansari, Ajay Kumar, Stanisław Fic, Danuta Barnat-Hunek

**Affiliations:** 1 Department of Civil Engineering, National Institute of Technology, Patna, Patna-800005, India; er.mdirfan2k9@gmail.com; 2 Faculty of Civil Engineering and Architecture, Lublin University of Technology, 40 Nadbystrzycka Str., 20-618 Lublin, Poland; s.fic@pollub.pl (S.F.); d.barnat-hunek@pollub.pl (D.B.-H.)

**Keywords:** functionally graded material, carbon nanotube, cubic variation of thickness co-ordinate, finite element method, bending, free vibration, concentrated mass

## Abstract

This paper examines the effect of uniaxially aligned carbon nanotube (CNT) on flexural and free vibration analysis of CNT-reinforced functionally graded plate. The mathematical model includes expansion of Taylor’s series up to the third degree in the thickness co-ordinate. Since there is a parabolic variation in transverse shear strain deformation across the thickness co-ordinate, the shear correction factor is not necessary. A nine-node two-dimensional (2D) *C*^0^ isoparametric element containing seven nodal unknowns per node was developed in the finite element code. The final material properties of CNT-reinforced functionally graded plate are estimated using the extended rule of mixture. The effect of CNT distribution, boundary condition, volume fraction and loading pattern are studied by developing a finite element code. An additional finite element code was developed for the study of the influence of concentrated mass on free vibration analysis of CNT-reinforced functionally graded plate.

## 1. Introduction

In the modern age, carbon nanotube (CNT)-reinforced composite plates have found considerable application in civil, mechanical, aeronautical and marine engineering due to their exceptional mechanical, thermal and electrical properties. The high tensile properties of CNT make CNT-reinforced composites preferable in tension-dominated applications such as pressure vessels. The concentrated mass is generally used to reduce the fundamental frequency to the desired value. The CNTs are allotropes of carbon having a length scale in the order of nanometres discovered by Iijima [1], having higher strength/weight ratio and lower density. Due to their superior properties, the CNTs are substantially preferable as a reinforcing choice for advanced composites. The Eshelby-Mori-Tanaka approach and a 2-D generalised differential quadrature method was used by Aragh et al. [2] for the frequency analysis of continuously graded CNT-reinforced cylindrical panel. The effect of singly walled carbon nanotubes (SWCNTs) on bending and vibration analysis of CNT-reinforced functionally graded (FG-CNT) plate was studied by Zhu et al. [3] with the help of the finite element method. Their mathematical model is based on the first order shear deformation theory (FSDT). Yas et al. [4] developed a three-dimensional model to study the vibration behaviour of functionally graded cylindrical panel reinforced with CNT. The element-free kp-Ritz method was used by Lei et al. [5] to study the free vibration analysis of CNT-reinforced composite (CNTRC) plate assuming an FSDT based displacement field. The deflection and stresses developed in CNT-reinforced composite cylinders have been studied by Dastjerdi et al. [6] using the mesh-free method. The FSDT-based displacement model was adopted by Zhang et al. [7] to analyse the flexural and free vibration response of CNT-reinforced composite panel. The Eshelby–Mori–Tanaka approached was used by them to calculate the final properties of CNT-reinforced cylindrical panel. The ending behaviour of FG-CNTRC cylindrical shell under mechanical loading was studied by Mehrabadi and Aragh [8]. They also incorporated the Eshelby–Mori–Tanaka approach, to calculate the effective material properties of uniformly distributed (UD) and FG-CNT-reinforced cylindrical shell. Budarapu et al. [9] developed a method to calculate the natural frequencies of multi-walled CNT embedded in an elastic medium. The higher order shear deformation theory (HSDT) is used by Sankar et al. [10] to study the static and free vibrations of FG-CNTRC plates and sandwich plates. Nami and Janghorban [11] used a three-dimensional elastic theory to analyse the free vibration behaviour of FG-CNTRC plate. Zhang et al. [12] explored the behaviour of CNT-reinforced plate with elastically restrained edges, using the element-free Ritz method incorporating FSDT, while Macias et al. [13] used FSDT along with a four-noded shell element for the investigation of CNT-reinforced functionally graded skew plate. Zhang and Selim [14] and Selim et al. [15] have both used Reddy’s HSDT displacement field for the dynamics analyses of FG-CNT-reinforced composite plate. The vibration analysis of doubly curved composite shell panel reinforced with CNT was studied by Pouresmaeell and Fzelzadeh [16]. Tornabene et al. [17] and Fantuzzi [18] adopted a micro-mechanical model for the study of dynamic behaviour of FG-CNT-reinforced arbitrary shaped plate and shell. They used Non-Uniform Rational B-Splines (NURBS) curves to obtain the arbitrary shape. Banic et al. [19] explored the vibration behaviour of composite plate and shell, reinforced with agglomerated CNT, which rested on Winkler–Pasternak elastic foundation. The mechanical properties are estimated using a modified rule of mixture. The non-linear thermo-elastic frequency analysis of CNT-reinforced functionally graded single and doubly curved shell has been carried out by Mehar et al. [20]. The FSDT was used by Huang et al. [21] to study the bending and free vibration behaviour of laminated CNT-reinforced plate. They have used the extended rule of mixture to compute the effective properties of material and adopted four-variable theories for a mathematical model. Asadi et al. [22] discussed the aero-thermo-elastic behaviour of supersonic FG-CNTRC flat panel in a thermal environment. The model is based on the FSDT incorporated with the von Karman geometric non-linearity. The experimental, numerical and simulation model for deflection behaviour of CNTRC plate was developed by Mehar and Panda [23]. Demirbas [24] developed an elastic theory for thermal analysis of functionally graded material (FGM) plate subjected to in-plane constant heat flux. Tornabene et al. [25] used FSDT and the generalised differential quadrature method to analyse the free vibration behaviour of laminated nano-composite plate and shell. They modelled each layer of the laminate as a three-phase composite. Size-dependent analysis of functionally graded microplate by using isogeometric analysis is studied by Liu et al. [26,27].

The static and free vibration analysis of an FG-CNT-reinforced plate will be complex using elastic solution or analytical method [15,28,29,30,31]. Apart from this, the elastic and analytical solutions are more difficult to obtain for complex boundary conditions. Therefore, in this paper, an effort has been made for the behavioural study of the CNT-reinforced functionally graded plate for various combinations of end support using third order shear deformation theory (TSDT), which omit the necessity of the shear correction factor. To the best of authors knowledge, no work has been done on flexural and free vibration analysis of FG-CNT-reinforced plate using 2D *C*^0^ finite element (FE) model using TSDT. In present analysis 2D *C*^0^ model is adopted along with finite element method which are more convenient due to the low computational effort requirement. The effective material properties of FG-CNT-reinforced plates are estimated using the extended rule of mixture. Three FE coding (static analysis, free vibration analysis and free vibration analysis with concentrated mass) were developed by the authors for the current model. Since there are no available results in the literature for the bending of FG-CNT-reinforced composite plates subjected to trigonometrical loading, and free vibration analysis of FG-CNT-reinforced composite plate with concentrated mass, hence the present analyses results may be useful for scholars working in this field. The mode shapes of CNT-reinforced plates are also plotted using MATLAB coding (MathWorks, Natick, MA, USA).

## 2. Effective Material of CNT-reinforced Functionally Graded Plates

**Figure 1:**
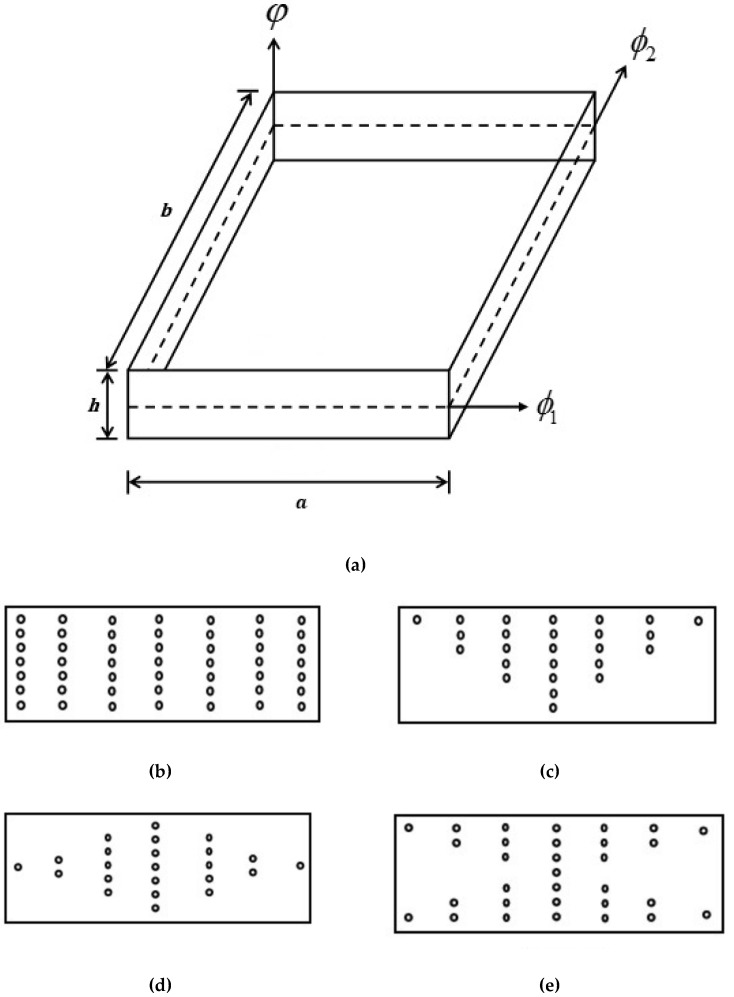
Configuration of carbon nanotube reinforced functionally graded plates. (**a**) Geometry of CNT-reinforced plate; (**b**) UD; (**c**) FG-V; (**d**) FG-O; (**e**) FG-X.

In the present analysis, the geometry of CNT-reinforced plates is depicted in Figure 1 and is referred to the $\left(\phi_{1},\;\phi_{2},\;\varphi \right)$ co-ordinates system. The FG-CNT-reinforced plate has a constant thickness *h*, with the length of the plate *a*, and width *b*. In this work, three types of functionally graded distribution (FG-O, FG-X and FG-V) and uniformly distributed (UD) of SWCNTs in polymer matrix across the thickness direction is considered. The extended rule of mixture [32,33], which contains the efficiency parameters, is incorporated for the calculation of effective material properties of the FG-CNT-reinforced composite plate.

(1) $$E_{11}=\eta_{1}V_{\mathrm{CNT}}E_{11}^{\mathrm{CNT}}+V_{m}E^{m}$$

(2) $$\frac{\eta_{2}}{E_{22}}=\frac{V_{\mathrm{CNT}}}{E_{22}^{\mathrm{CNT}}}+\frac{V_{m}}{E^{m}}$$

(3) $$\frac{\eta_{3}}{G_{12}}=\frac{V_{\mathrm{CNT}}}{G_{12}^{\mathrm{CNT}}}+\frac{V_{m}}{G^{m}}$$

(4) $$\nu_{12}=V_{\mathrm{CNT}}^{*}\nu_{12}^{\mathrm{CNT}}+V_{m}\nu^{m}$$

(5) $$\rho_{12}=V_{\mathrm{CNT}}^{*}\rho^{\mathrm{CNT}}+V_{m}\rho^{m}$$

where $\left(E_{11}^{\mathrm{CNT}},\;E_{22}^{\mathrm{CNT}},\;G_{12}^{\mathrm{CNT}}\right)$ are Young’s modulus and shear modulus of SWCNTs, respectively. The notations $\left(E^{m},\;G^{m}\right)$ are known as Young’s modulus and shear modulus of the polymer matrix. The CNT efficiency parameter $\left(\eta_{1},\;\eta_{2},\;\eta_{3}\right)$ are the scale-dependent material properties. $\left(\nu^{m},\;\rho^{m}\right)$ and $\left(\nu_{12}^{\mathrm{CNT}},\;\rho^{\mathrm{CNT}}\right)$ represents the Poisson’s ratio and mass density of matrix and SWCNT, respectively. The volume fractions of the SWCNT and matrix are denoted by $V_{\mathrm{CNT}}\;\mathrm{and}\;V_{m}$, respectively, and their additions are equal to unity. 

The volume fraction of CNTs as a function of the thickness co-ordinate can be expressed as [32,33]:

(6) $$V_{\mathrm{CNT}}\left(\varphi \right)=\left\{ \begin{array}{ll} V_{\mathrm{CNT}}^{*} & \left(\mathrm{UD}\right)\\ 2\left(1-\frac{2\left|\varphi \right|}{h}\right)V_{\mathrm{CNT}}^{*} & \left(\mathrm{FG-O}\right)\\ 2\left(\frac{2\left|\varphi \right|}{h}\right)V_{\mathrm{CNT}}^{*} & \left(\mathrm{FG-X}\right)\\ \left(1+\frac{2\varphi }{h}\right)V_{\mathrm{CNT}}^{*} & \left(\mathrm{FG-V}\right) \end{array}\right.$$

where $V_{\mathrm{CNT}}^{*}=\frac{w_{\mathrm{CNT}}}{w_{\mathrm{CNT}}+\left(\rho^{\mathrm{CNT}}/\rho^{m}\right)-\left(\rho^{\mathrm{CNT}}/\rho^{m}\right)w_{\mathrm{CNT}}}$, $w_{\mathrm{CNT}}$ denoted the mass fraction of the CNTs inside a CNT-reinforced plate. $\rho^{\mathrm{CNT}}$ and $\rho^{m}$ are densities of the carbon nanotubes and matrix, respectively.

## 3. Theoretical Formulation

### 3.1. Displacement Fields and Strains

Based on the third-order shear deformation theory, the displacement field (*u*,*v*,*w*) can be determined as follows [34]:

(7) $$u\left(\phi_{1},\phi_{2},\varphi \right)=u_{0}\left(\phi_{1},\phi_{2}\right)+\varphi \theta_{1}\left(\phi_{1},\phi_{2}\right)+\varphi^{2}\xi_{1}\left(\phi_{1},\phi_{2}\right)+\varphi^{3}\zeta_{1}\left(\phi_{1},\phi_{2}\right)\;v\left(\phi_{1},\phi_{2},\varphi \right)=v_{0}\left(\phi_{1},\phi_{2}\right)+\varphi \theta_{2}\left(\phi_{1},\phi_{2}\right)+\varphi^{2}\xi_{2}\left(\phi_{1},\phi_{2}\right)+\varphi^{3}\zeta_{2}\left(\phi_{1},\phi_{2}\right)\;w\left(\phi_{1},\phi_{2},\varphi \right)=w_{0}\left(\phi_{1},\phi_{2}\right)$$

where $\left(u_{0},\;v_{0},\;w_{0}\right)$ are the displacements along the $\left(\phi_{1},\;\phi_{2},\;\varphi \right)$ directions, respectively, at the mid-plane (φ=0). $\left(\theta_{1},\;\theta_{2}\right)$ are the bending rotations about the $\phi_{2}$ and $\phi_{1}$ axes, respectively. $\left(\xi_{1},\;\xi_{2},\;\zeta_{1},\;\zeta_{2}\right)$ are known as the higher order terms of Taylor’s series expansion. The unknown terms $\left(\xi_{1},\;\xi_{2},\;\zeta_{1},\;\zeta_{2}\right)$ are computed by applying zero shear stress at the lower and upper surfaces of a CNT-reinforced plate. Utilising the boundary conditions $\gamma_{\phi_{1}\phi_{2}}\left(\phi_{1},\;\phi_{2},\;\pm h/2\right)=\gamma_{\phi_{1}\phi_{2}}\left(\phi_{1},\;\phi_{2},\;\pm h/2\right)=0$ at the top and bottom surfaces of the plate in Equation (7), we obtained Taylor’s series expansion terms as

(8) $$\xi_{1}=\xi_{2}=0$$

(9) $$\zeta_{1}=-\frac{4}{3h^{2}}\left(\theta_{1}+\frac{\partial w}{\partial \phi_{1}}\right),\;\zeta_{2}=-\frac{4}{3h^{2}}\left(\theta_{2}+\frac{\partial w}{\partial \phi_{2}}\right)$$

Substituting Equation (8) into Equation (7), we obtain

(10) $$u\left(\phi_{1},\phi_{2},\varphi \right)=u_{0}+\left(\varphi -\frac{4\varphi^{3}}{3h^{2}}\right)\theta_{1}-\frac{4\varphi^{3}}{3h^{2}}\left(\frac{\partial w}{\partial \phi_{1}}\right)\;v\left(\phi_{1},\phi_{2},\varphi \right)=v_{0}+\left(\varphi -\frac{4\varphi^{3}}{3h^{2}}\right)\theta_{2}-\frac{4\varphi^{3}}{3h^{2}}\left(\frac{\partial w}{\partial \phi_{2}}\right)\;w\left(\phi_{1},\phi_{2},\varphi \right)=w_{0}$$

During the implementation of the displacement field represented in Equation (10), the problem of *C*^1^ continuity is encountered due to the presence of first order derivatives of the transverse displacement component in the expression of in-plane fields. For applying efficient *C*^0^ FE formulation, the derivatives are replaced by the appropriate substitution of an independent nodal unknowns as

(11) $$\psi_{1}=\frac{\partial w}{\partial \phi_{1}},\;\psi_{2}=\frac{\partial w}{\partial \phi_{2}}$$

The higher order displacement field owning *C*^0^ continuity can express as:

(12) $$u\left(\phi_{1},\phi_{2},\varphi \right)=u_{0}+\left(\varphi -\frac{4\varphi^{3}}{3h^{2}}\right)\theta_{1}-\frac{4\varphi^{3}}{3h^{2}}\psi_{1}\;v\left(\phi_{1},\phi_{2},\varphi \right)=v_{0}+\left(\varphi -\frac{4\varphi^{3}}{3h^{2}}\right)\theta_{2}-\frac{4\varphi^{3}}{3h^{2}}\psi_{2}\;w\left(\phi_{1},\phi_{2},\varphi \right)=w_{0}$$

Hence, the degree of freedom (basic field variables) according to present mathematical formulation for each node is

(13) $$\left\{\delta \right\}={\left[u_{0},\;v_{0},\;w_{0},\;\theta_{1},\;\theta_{2},\;\psi_{1},\;\psi_{2}\right]}^{T}$$

where {δ} is named as the displacement vector.

The strain vector from the above displacement field can be written as

(14) $$\left\{\varepsilon \right\}={\left\{\varepsilon_{1},\;\varepsilon_{2},\;\varepsilon_{6},\;\varepsilon_{4},\;\varepsilon_{5}\right\}}^{T}$$

Further, the relations between the strain vector {ε} and the displacement vector {δ} can be expressed as

(15) {ε}=[B]{δ}

where the strain-displacement matrix [*B*] contains the derivatives of shape function.

The in-plane and transverse shear strains are

(16) $$\varepsilon_{1}=\varepsilon_{\phi_{1}\phi_{1}}=\frac{\partial u}{\partial \phi_{1}}\;\varepsilon_{2}=\varepsilon_{\phi_{2}\phi_{2}}=\frac{\partial v}{\partial \phi_{2}}\;\varepsilon_{6}=\gamma_{\phi_{1}\phi_{2}}=\frac{\partial u}{\partial \phi_{2}}+\frac{\partial v}{\partial \phi_{1}}\;\varepsilon_{4}=\gamma_{\phi_{1}\varphi }=\frac{\partial u}{\partial \varphi }+\frac{\partial w}{\partial \phi_{1}}\;\varepsilon_{5}=\gamma_{\phi_{2}\varphi }=\frac{\partial v}{\partial \varphi }+\frac{\partial w}{\partial \phi_{2}}$$

The strain relationships can be written as

(17) $$\varepsilon_{1}=\varepsilon_{1}^{0}+\varphi k_{1}^{1}+\varphi^{3}k_{1}^{3}\;\varepsilon_{2}=\varepsilon_{2}^{0}+\varphi k_{2}^{1}+\varphi^{3}k_{2}^{3}\;\varepsilon_{6}=\varepsilon_{6}^{0}+\varphi k_{6}^{1}+\varphi^{3}k_{6}^{3}\;\varepsilon_{4}=\varepsilon_{4}^{0}+\varphi^{2}k_{4}^{2}\;\varepsilon_{5}=\varepsilon_{5}^{0}+\varphi^{2}k_{5}^{2}$$

where, $\varepsilon_{1}^{0}=\frac{\partial u_{0}}{\partial \phi_{1}}$, $\varepsilon_{2}^{0}=\frac{\partial v_{0}}{\partial \phi_{2}}$, $\varepsilon_{6}^{0}=\frac{\partial u_{0}}{\partial \phi_{2}}+\frac{\partial v_{0}}{\partial \phi_{1}}$, $\varepsilon_{4}^{0}=\frac{\partial w_{0}}{\partial \phi_{2}}$, $\varepsilon_{5}^{0}=\frac{\partial w_{0}}{\partial \phi_{1}}$, $k_{1}^{1}=\frac{\partial \theta_{1}}{\partial \phi_{1}}$, $k_{2}^{1}=\frac{\partial \theta_{2}}{\partial \phi_{2}}$, $k_{6}^{1}=\frac{\partial \theta_{1}}{\partial \phi_{2}}+\frac{\partial \theta_{2}}{\partial \phi_{1}}$, $k_{6}^{3}=-\frac{4}{3h^{2}}\left(\left(\frac{\partial \theta_{1}}{\partial \phi_{2}}+\frac{\partial \psi_{1}}{\partial \phi_{2}}\right)+\left(\frac{\partial \theta_{2}}{\partial \phi_{1}}+\frac{\partial \psi_{2}}{\partial \phi_{1}}\right)\right)$, $k_{1}^{3}=-\frac{4}{3h^{2}}\left(\frac{\partial \theta_{1}}{\partial \phi_{1}}+\frac{\partial \psi_{1}}{\partial \phi_{1}}\right)$, $k_{2}^{3}=-\frac{4}{3h^{2}}\left(\frac{\partial \theta_{2}}{\partial \phi_{2}}+\frac{\partial \psi_{2}}{\partial \phi_{2}}\right)$, $k_{4}^{2}=\left(\left(1-\frac{4}{h^{2}}\right)\theta_{1}-\frac{4}{h^{2}}\psi_{1}\right)$, $k_{5}^{2}=\left(\left(1-\frac{4}{h^{2}}\right)\theta_{2}-\frac{4}{h^{2}}\psi_{2}\right)$.

### 3.2. Constitutive Relations

The linear stress-strain constitutive relationships for the CNT-reinforced plate are

(18) {σ}=[Q]{ε}

where the constitutive matrix

(19) $$\left[Q\right]=\left[\begin{array}{lllll} Q_{11} & Q_{12} & 0 & 0 & 0\\ Q_{21} & Q_{22} & 0 & 0 & 0\\ 0 & 0 & Q_{33} & 0 & 0\\ 0 & 0 & 0 & Q_{44} & 0\\ 0 & 0 & 0 & 0 & Q_{55} \end{array}\right]$$

The term $Q_{ij}$ can be obtained from the material properties which are the function of the depth of the plate.

(20) $$Q_{11}=\frac{E_{11}}{1-\nu_{12}\nu_{21}},\;Q_{22}=\frac{E_{22}}{1-\nu_{12}\nu_{21}},\;Q_{12}=\frac{\nu_{21}E_{11}}{1-\nu_{12}\nu_{21}},\;Q_{33}=Q_{44}=Q_{55}=G_{12}$$

## 4. Finite Element Method

### 4.1. Element Description

For the present *C*^0^ finite element (FE) model, nine-node isoparametric Lagrangian elements with node-wise seven degrees of freedom are employed. The shape function (interpolation function) is used to express the generalised displacement vector and element geometry at any point within an element as:

(21) $$\left\{\delta \right\}=\sum_{i=1}^{9}N_{i}\left(\xi ,\eta \right){\left\{\delta \right\}}_{i}\;\left\{\phi_{1}\right\}=\sum_{i=1}^{9}N_{i}\left(\xi ,\eta \right){\left\{\phi_{1}\right\}}_{i}\;\left\{\phi_{2}\right\}=\sum_{i=1}^{9}N_{i}\left(\xi ,\eta \right){\left\{\phi_{2}\right\}}_{i}$$

where *N_i_* is the shape function of nine-node isoparametric Lagrangian elements [35].

### 4.2. Flexural Analysis

The strain energy may be expressed as

(22) $$U=\frac{1}{2}∭{\left\{\varepsilon \right\}}^{T}\left\{\sigma \right\}d\phi_{1}d\phi_{2}d\varphi$$

By using the Equation (18), the above expression can be represented as

(23) $$U=\frac{1}{2}\iint {\left\{\varepsilon \right\}}^{T}\left[D\right]\left\{\varepsilon \right\}d\phi_{1}d\phi_{2}$$

where $\left[D\right]={\int \left[H\right]}^{T}\left[Q\right]\left[H\right]d\varphi$ in which [*H*] matrix contains *φ* and *h*.

The global stiffness matrix is derived by minimising the total energy of the CNT-reinforced plate as

(24) $$\left[K\right]={\iint \left[B\right]}^{T}\left[D\right]\left[B\right]d\phi_{1}d\phi_{2}$$

By using the standard procedure, the FE equations of CNT-reinforced plates subjected to transverse load can be expressed as

(25) [K]{δ}={F}

where {F} and [K] are load vector and global stiffness matrix.

### 4.3. Free Vibration Analysis

The governing equation of free vibration analysis of CNT-reinforced plates is expressed as

(26) $$\left(\left[K\right]-\omega^{2}\left[M\right]\right)\left\{X\right\}=\left\{0\right\}$$

where [*K*] and [*M*] are the global stiffness matrix and global mass matrix, respectively. The global stiffness matrix [*K*] is the same as expressed in Equation (24).

The element mass matrix shown below is derived by applying Hamilton’s principle.

(27) $$\left[m\right]=\iint_{A}{\left[C\right]}^{T}\left[L\right]\left[C\right]d\phi_{1}\phi_{2}$$

where matrix [*C*] matrix contains shape function (*N_i_*).

The [*L*] matrix can be stated as:

(28) $$\left[L\right]={\int_{\varphi }\rho \left[F\right]}^{T}\left[F\right]d\varphi$$

where the matrix [*F*] of order 3 × 7 contains *φ* and some constant quantities like that of [*H*] and *ρ* is known as the density which will be calculated from Equation (5). 

## 5. Numerical Result and Discussion

In this section, many numerical examples were studied for the flexural and free vibration behaviour of CNT-reinforced functionally grade plates. PmPV [36] was for the matrix and for reinforcing the material armchair (10,10) SWCNTs were chosen. The material properties of SWCNT and the matrix at room temperature (300 K) are given as

$$E_{11}^{\mathrm{CNT}}=5.6466\;\mathrm{TPa},\;E_{22}^{\mathrm{CNT}}=7.08\;\mathrm{TPa},\;G_{12}^{\mathrm{CNT}}=1.9445\;\mathrm{TPa},\;\nu_{12}^{\mathrm{CNT}}=0.175,\;\rho^{\mathrm{CNT}}=1400\;\mathrm{kg}/m^{3}\;E^{m}=2.1\;\mathrm{GPa},\;\nu^{m}=0.34,\;\rho^{m}=1150\;\mathrm{kg}/m^{3}$$

The CNT efficiency parameters for considered three types of volume fraction are given as:

$$\mathrm{For}\;V_{\mathrm{CNT}}^{*}=0.11;\;\eta_{1}=0.149,\;\eta_{2}=0.934,\;\eta_{3}=0.934\;\mathrm{For}\;V_{\mathrm{CNT}}^{*}=0.11;\;\eta_{1}=0.150,\;\eta_{2}=0.941,\;\eta_{3}=0.941\;\mathrm{For}\;V_{\mathrm{CNT}}^{*}=0.11;\;\eta_{1}=0.149,\;\eta_{2}=1.381,\;\eta_{3}=1.381$$

The quantities used in the present study are:

For the flexural analysis

$$\bar{w}=\frac{wE^{m}h^{3}}{q_{0}a^{4}},\;{\bar{\sigma }}_{\phi_{1}\phi_{1}}=\sigma_{\phi_{1}\phi_{1}}\left(\frac{a}{2},\frac{b}{2},\varphi \right)\frac{h^{2}}{q_{0}a^{2}}$$

For the free vibration analysis

$$\bar{\omega }=\omega \left(a^{2}/h\right)\sqrt{\rho^{m}/E^{m}}$$

Concentrated mass

$$\bar{M}=M/\rho^{m}a^{2}h$$

The loading patterns are used as:

$$q=q_{0},\;q=q_{0}\sin\left(\frac{\pi \phi_{1}}{a}\right)\sin\left(\frac{\pi \phi_{2}}{b}\right)\;q=q_{0}\cos\left(\frac{\pi \phi_{1}}{a}\right)\sin\left(\frac{\pi \phi_{2}}{b}\right),\;q=q_{0}\cos\left(\frac{\pi \phi_{1}}{a}\right)\cos\left(\frac{\pi \phi_{2}}{b}\right)$$

The details of end support conditions used in the present study are:

1. Clamped (CCCC):

$$\mathrm{At}\;\phi_{1}=0,a\;\mathrm{and}\;\phi_{2}=0,b\;u=v=w=\theta_{\phi_{1}}=\theta_{\phi_{2}}=\psi_{\phi_{1}}=\psi_{\phi_{2}}=0$$

2. Simply supported (SSSS):

$$\mathrm{At}\;\phi_{1}=0,a\;v=w=\theta_{\phi_{2}}=\psi_{\phi_{2}}=0\;\mathrm{At}\;\phi_{2}=0,b\;u=w=\theta_{\phi_{1}}=\psi_{\phi_{1}}=0$$

3. Clamped and simply supported (CCSS):

$$\mathrm{At}\;\phi_{1}=0,a\;u=v=w=\theta_{\phi_{1}}=\theta_{\phi_{2}}=\psi_{\phi_{1}}=\psi_{\phi_{2}}=0\;\mathrm{At}\;\phi_{2}=0,b\;u=w=\theta_{\phi_{1}}=\psi_{\phi_{1}}=0$$

### Convergence and Validation Study

To check the suitable number of mesh sizes to attain precise results, a convergence study was performed for both flexural and free vibration analyses of CNT-reinforced functionally graded plates. Table 1 and Table 2 show the convergence study for the fundamental frequency and deflection of a clamped FG-CNT-reinforced plate. The results are computed for $V_{\mathrm{CNT}}^{*}=0.11$ and *a/h* = 10 for different mesh sizes. These convergence studies highlighted that for free vibration analysis and bending analysis of FG-CNT-reinforced plates, a 16 × 16 mesh size is satisfactory. Table 3 shows the results of the free vibration analyses for an isotropic square plate (*ν* = 0.3). The dimensionless frequency parameter of the isotropic plate was compared with HSDT results for a moderately thick plate [37] and an exact solution [38]. For more investigation, a detailed comparison has been done for free vibration and bending analyses considering three thickness ratios (*a/h* = 10, 20 and 50) and three volume fractions $\left(V_{\mathrm{CNT}}^{*}=0.11,\;0.14\;\mathrm{and}\;0.17\right)$. The calculated frequency parameter shown in Table 4 and Table 5 for simply supported boundary conditions are in line with previous result provided by Zhu et al. [3]. Table 6 shows the central deflection of the UD reinforced composite plate for CCCC, SSSS, SCSC and SFSF boundary conditions. Our numerical results confirm with previous result given by Zhu et al. [3]. 

Afterwards, the parametric studies have been conducted to examine the effect of boundary conditions (SSSS, CCCC, CCSS, CSCS, CCFF and CFCF), thickness ratios (*a/h*), concentrated mass, as well as, the volume fraction of CNT $\left(V_{\mathrm{CNT}}^{*}\right)$ on the flexural and free vibration behaviour of CNT-reinforced functionally graded plate. The non-dimensional frequency of the first six modes for FG-CNT-reinforced plate is presented in Table 7, Table 8 and Table 9 for the three-different types of $V_{\mathrm{CNT}}^{*}=0.11,\;0.14\;\mathrm{and}\;0.17$, respectively. The results are computed for *a/b* = 1 and *a/h* = 10. For the all considered boundary conditions, minimum and maximum non-dimensional frequency parameters were noted for FG-O and FG-X distribution among the other considered distribution. Rather than mid-section, the top and bottom section of the plate was chosen for the distribution of additional CNT to achieve maximum stiffness. Thus, the FG-O and FG-X distributions produce minimum and maximum stiffness, respectively. Further, it was also noticed that CFCF yields minimum frequency parameters while the all side-clamped plate yields the maximum frequency parameter. This is because the higher constraints at the boundary give a higher stiffness to the CNT-reinforced functionally graded plate. Here, approximately, a 6% increase in non-dimensional fundamental frequency was noticed when the volume fraction of CNT increases from 0.11 to 0.14, around a 25% increase was noticed when $V_{\mathrm{CNT}}^{*}$ changes from 0.11 to 0.17. 

**Figure 2:**
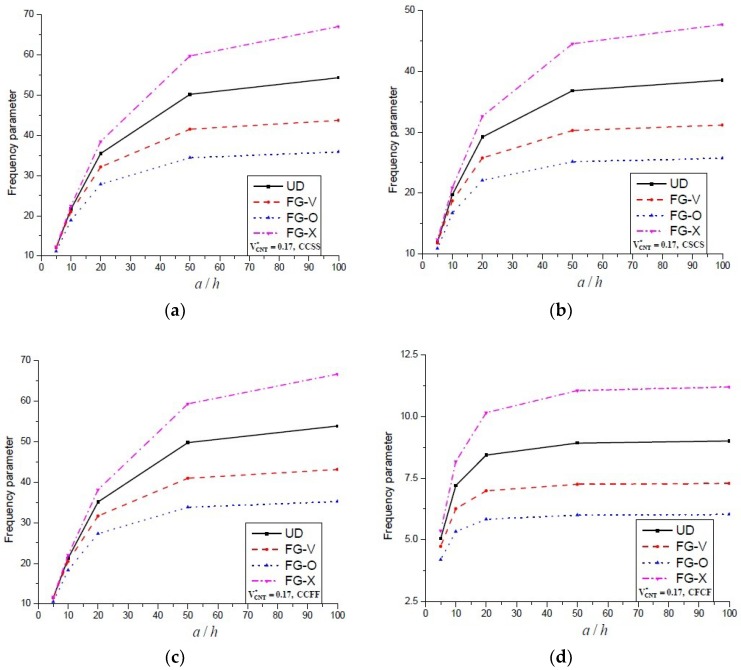
The variation of dimensionless frequency parameter vs. *a/h* ratio for an FG-CNT-reinforced plate with different types of boundary conditions. (**a**) CCSS; (**b**) CSCS; (**c**) CCFF and (**d**) CFCF.

Figure 2 shows the effect of side-to-thickness ratio on the non-dimensional fundamental frequency of FG-CNT-reinforced plates. The results are calculated for $V_{\mathrm{CNT}}^{*}$ = 0.17 for CCSS, CSCS, CCFF and CFCF boundary conditions. Here it can be seen that the dimensionless frequency parameters increase along with the *a/h* ratio and it became insensitive from *a/h* = 60 onwards for all used boundary conditions. The effect of the concentrated mass on the free vibrations of FG-CNT-reinforced plates, having simply supported boundary conditions, is presented in Table 10. It can be noticed that increases in concentrated mass at the centre decreases the fundamental frequency parameter while no significant reduction is seen for any other mode of frequencies. Here, an approximate 28% decrease in the fundamental frequency is noticed when the value of the concentrated mass is increased by 0.5–1 and 1–2. 

**Figure 3:**
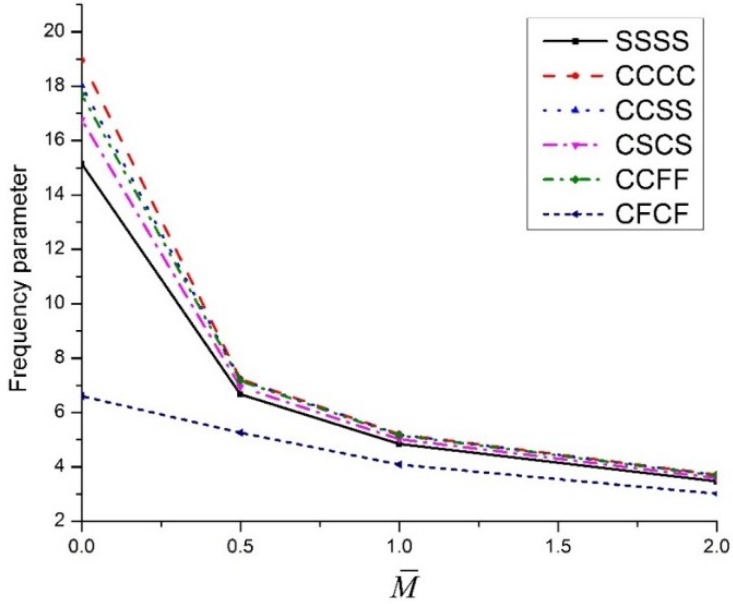
The variation of dimensionless frequency parameter vs. dimensionless concentrated mass for an FG-CNT-reinforced plate for various boundary conditions.

**Figure 4:**
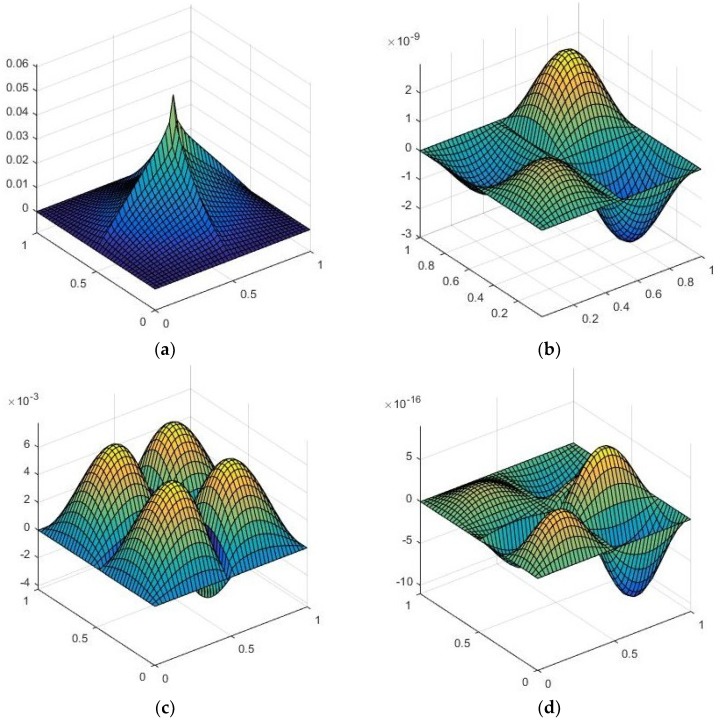
The first four mode shape of a UD-CNT-reinforced square plate with concentrated mass $\left(\bar{M}=1\right)$ at the centre for $\left(V_{\mathrm{CNT}}^{*}=0.17\right)$. (**a**) Mode 1; (**b**) Mode 2; (**c**) Mode 3 and (**d**) Mode 4.

Figure 3 shows the effect of concentrated mass on the vibration behaviour of an FG-CNTRC plate having various types of boundary conditions. For all considered boundary conditions, the dimensionless frequency parameter decreases, with an increase in the concentrated mass; and the CFCF boundary conditions have the least effect of concentration among considered boundary conditions. The first mode shape of a UD-CNT-reinforced plate, with concentrated mass at the centre, is presented in Figure 4.

The maximum deflection of an FG-CNT-reinforced plate having various side-to-thickness ratios for $V_{\mathrm{CNT}}^{*}=0.11,\;0.14\;\mathrm{and}\;0.17$ subjected to sin-sin loading are presented in Table 11, Table 12 and Table 13, respectively. The results are calculated for UD, FG-V, FG-O and FG-X distribution of CNT across the transverse direction, having an aspect ratio *a/b* = 1. A decrease in deflection is noted when the $V_{\mathrm{CNT}}^{*}$ increases because of the higher value of $V_{\mathrm{CNT}}^{*}$, imparts a higher stiffness in CNT-reinforced plate, thus the deflection is reduced. The maximum deflection decreases with an increase in the *a/h* ratio irrespective of boundary conditions and types of distribution. Our finding confirms that there is approximately a 7% reduction in the maximum deflection for all considered end support as the value of $V_{\mathrm{CNT}}^{*}$ increased from 0.11 to 0.14 and approximately a 36% decrease is found when $V_{\mathrm{CNT}}^{*}$ increases from 0.11 to 0.17. FG-X and FG-O distribution yields minimum and maximum deflection, respectively. 

**Figure 5:**
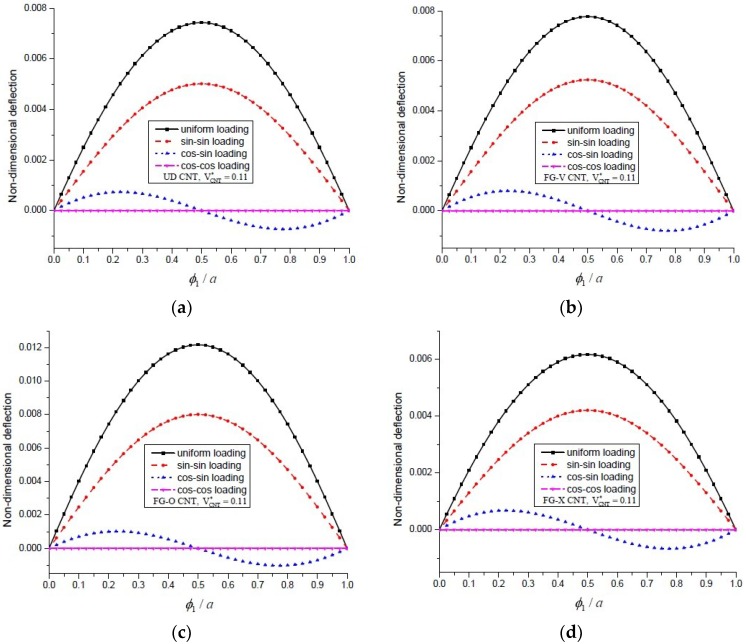
The variation of transverse deflection vs. the length $\phi_{1}/a$ for an FG-CNT-reinforced plate, for (**a**) UD; (**b**) FG-V; (**c**) FG-O and (**d**) FG-X distribution subjected to sin-sin loading.

Figure 5 shows the variation of deflection of UD, FG-V, FG-O and FG-X type CNT-reinforced plates along the centre line subject to the various types of mechanical load. The results are obtained for $V_{\mathrm{CNT}}^{*}=0.11$. It can be seen that, for all types of CNT distribution in the thickness direction, the graph of deflection along the length is of the same nature. 

**Figure 6:**
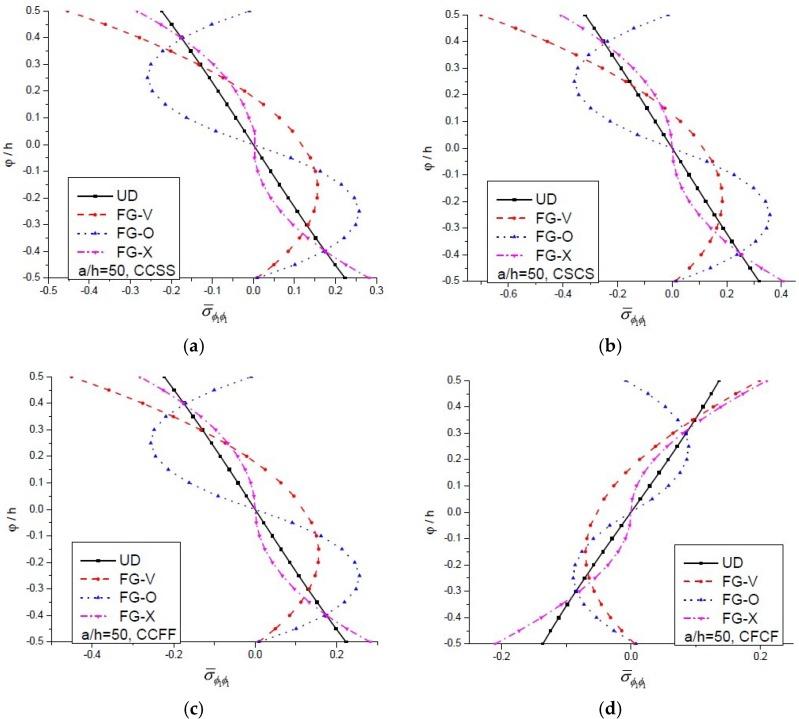
The deviation of dimensionless axial stress $\left({\bar{\sigma }}_{\phi_{1}\phi_{1}}\right)$ vs. φ/h ratio for an FG-CNT-reinforced plate under sin-sin loading, for (**a**) CCSS; (**b**) CSCS; (**c**) CCFF and (**d**) CFCF boundary conditions.

The minimum and maximum deflections were noticed for cos-cos type of loading and uniform loading, respectively. The axial stress developed in a CNT-reinforced functionally graded plate under sin-sin loading is plotted in Figure 6 against the thickness co-ordinate for CCSS, CSCS, CCFF and CFCF support conditions. The non-dimensional axial stress decreases with an increase in constraints at end support. It is interesting to note that for all types of boundary conditions, except CFCF, the nature of the graph along thickness co-ordinate is the same, for CFCF type boundary conditions, the nature of the graph is opposite to other taken boundary conditions.

## 6. Conclusions

In the present work, a *C*^0^ FE model based on Reddy’s TSDT was developed to investigate the flexural and free vibration behaviour of CNT-reinforced functionally graded plates. The CNT distribution through the thickness of plate is assumed to be uniform or functionally graded. The properties of CNT-reinforced plates at any point are calculated using the modified rule of mixture in which efficiency parameters are introduced into the rule of mixtures approach. The influence of the concentrated mass, volume fraction, side-to-thickness ratios, loading pattern and end support condition on the dimensionless bending and frequency parameter were also studied. Based on the present results, it can be concluded that:

- Among the considered distribution pattern of CNT, FG-X pattern results in higher dimensionless frequency parameter and lower deflection, while FG-O pattern yields lower dimensionless frequency parameters and higher dimensionless deflections.
- An increase in the dimensionless frequency parameters and decrease in the deflection of FG-CNT-reinforced plate is found when the volume fraction of CNT is increased.
- With the increase in side-to-thickness ratio, an increase in dimensionless frequency and a decrease in deflection is noticed.
- The greater constraints on boundaries results in lower values of deflection and higher values of dimensionless frequency parameters.
- The concentrated mass at the centre decreases the fundamental frequency parameter.

## Figures and Tables

**Table 1:** Convergence study of the present results for the dimensionless frequency parameter of a CNT-reinforced plate for clamped boundary conditions.

Mesh Size	UD	FG-V	FG-O	FG-X
8 × 8	18.2872	17.7565	16.0741	18.9550
10 × 10	18.2860	17.7554	16.0728	18.9538
12 × 12	18.2848	17.7542	16.0719	18.9531
14 × 14	18.2843	17.7536	16.0714	18.9526
16 × 16	18.2842	17.7534	16.0716	18.9525

**Table 2:** Convergence study of the present results for the deflection of a CNT-reinforced plate for clamped boundary conditions.

Mesh Size	UD	FG-V	FG-O	FG-X
8 × 8	0.00904	0.00926	0.01061	0.00867
10 × 10	0.00892	0.00918	0.01049	0.00856
12 × 12	0.00884	0.00914	0.01044	0.00851
14 × 14	0.00881	0.00912	0.01041	0.00848
16 × 16	0.00880	0.00912	0.01040	0.00848

**Table 3:** Dimensional frequency parameter of the simply supported square isotropic plate.

Reference	Mode		
	(1,1)	(1,2)	(1,3)
Present	0.093	0.221	0.415
Mantari et al. [37]	0.093	0.222	0.415
Srinivas et al. [38]	0.093	0.223	0.417

**Table 4:** Dimensionless first six natural frequencies $\bar{\omega }$ for a UD CNT-reinforced square plate with *a/h* ratios.

$V_{\mathrm{CNT}}^{*}$	Mode	*a/h* = 10		*a/h* = 20		*a/h* = 50	
		Ref. [3]	Present	Ref. [3]	Present	Ref. [3]	Present
0.11	1	17.625	18.284	28.400	29.232	39.730	41.246
	2	23.041	23.793	33.114	34.108	43.876	45.501
	3	33.592	34.188	44.559	45.456	54.768	56.313
	4	33.729	35.188	59.198	60.708	74.488	75.080
	5	37.011	38.536	61.851	63.003	98.291	100.577
	6	37.317	38.738	63.043	63.553	100.537	101.437
0.14	1	18.127	18.854	29.911	30.795	43.583	45.216
	2	23.572	24.374	34.516	35.558	47.479	49.218
	3	34.252	34.874	45.898	46.830	57.968	59.617
	4	34.650	36.267	61.628	63.337	77.395	78.064
	5	37.921	39.384	64.199	64.457	106.371	104.359
	6	37.972	39.592	64.496	66.100	106.487	108.807
0.17	1	22.011	22.795	35.316	36.286	49.074	50.802
	2	28.801	29.679	41.253	42.400	54.324	56.170
	3	42.015	42.666	55.267	56.600	68.069	69.766
	4	42.132	43.878	73.769	75.518	92.868	93.286
	5	46.250	48.066	77.109	78.531	121.669	124.191
	6	46.694	48.343	78.801	79.084	124.518	126.244

**Table 5:** Dimensionless first six natural frequencies $\bar{\omega }$ for an FG-V CNT-reinforced square plate with *a/h* ratios.

$V_{\mathrm{CNT}}^{*}$	Mode	*a/h* = 10		*a/h* = 20		*a/h* = 50	
		Ref. [3]	Present	Ref. [3]	Present	Ref. [3]	Present
0.11	1	17.211	17.753	26.304	26.693	34.165	34.480
	2	22.812	23.462	31.496	32.099	39.043	39.584
	3	33.070	34.035	43.589	44.133	51.204	51.815
	4	33.552	34.355	56.249	57.061	72.202	71.954
	5	36.528	37.889	59.249	60.253	86.291	86.133
	6	37.437	38.841	62.608	62.218	89.054	89.105
0.14	1	17.791	18.405	27.926	28.371	37.568	37.909
	2	23.413	24.113	32.976	33.629	42.175	42.733
	3	34.101	34.792	44.989	45.573	53.963	54.590
	4	34.275	35.553	58.951	59.968	74.785	74.546
	5	37.538	39.053	61.816	63.051	94.022	93.911
	6	38.159	39.574	64.135	63.758	96.573	96.680
0.17	1	21.544	22.152	32.686	33.050	42.078	42.292
	2	28.613	29.332	39.279	39.895	48.309	48.796
	3	41.431	42.605	54.560	55.058	63.755	64.286
	4	42.119	42.912	70.149	70.903	90.293	89.657
	5	45.796	47.364	73.926	74.948	106.513	105.881
	6	47.055	48.721	78.522	77.777	110.055	109.679

**Table 6:** Maximum transverse deflection $\bar{w}$ for a UD CNT-reinforced square plate with *a/h* ratios.

BC	$V_{\mathrm{CNT}}^{*}$	*a/h* = 10		*a/h* = 20		*a/h* = 50	
		Ref. [3]	Present	Ref. [3]	Present	Ref. [3]	Present
CCCC	0.11	0.00222	0.00207	0.01339	0.01257	0.2618	0.24056
	0.14	0.00208	0.00192	0.01188	0.01115	0.2131	0.19644
	0.17	0.00141	0.00131	0.00856	0.00806	0.1698	0.15695
SSSS	0.11	0.00373	0.00354	0.03628	0.03352	1.1550	1.04729
	0.14	0.00330	0.00314	0.03001	0.02779	0.9175	0.83205
	0.17	0.00239	0.00227	0.02348	0.02180	0.7515	0.68655
SCSC	0.11	0.00332	0.00313	0.03393	0.03127	1.0990	0.99624
	0.14	0.00297	0.00281	0.02852	0.02634	0.8890	0.80555
	0.17	0.00212	0.00201	0.02190	0.02028	0.7135	0.65105
SFSF	0.11	0.00344	0.00339	0.03341	0.03223	1.0680	1.01428
	0.14	0.00302	0.00297	0.02760	0.02654	0.8505	0.80295
	0.17	0.00207	0.00218	0.02162	0.02096	0.6950	0.66441

**Table 7:** Dimensionless first six natural frequencies $\bar{\omega }$ for FG-CNT-reinforced plate with several types of boundary conditions $\left(V_{\mathrm{CNT}}^{*}=0.11,\;a/h=10\right)$.

CNT Distribution	Mode	SSSS	CCCC	CCSS	CSCS	CCFF	CFCF
UD CNT	1	13.8852	18.2842	17.3753	15.8868	17.0425	5.8250
	2	18.1994	23.7934	19.4223	20.8718	17.1008	8.9023
	3	19.4225	34.1882	20.6985	25.6174	18.5180	18.0312
	4	19.4275	35.1886	29.5574	31.1128	19.2125	19.2182
	5	28.1212	38.5362	34.7213	34.2514	24.5644	20.8003
	6	33.2913	38.7388	36.7252	36.978	34.4553	22.4299
FG-V CNT	1	12.6013	17.7534	16.8089	15.0616	16.4289	5.0825
	2	17.4092	23.4625	19.4794	20.3638	16.5145	8.5314
	3	19.4794	34.0359	20.3062	25.6864	18.0592	17.9081
	4	19.4848	34.3556	29.3712	30.8794	19.2392	19.1983
	5	27.7626	37.8893	33.8705	33.1631	24.3163	19.4767
	6	31.9032	38.8412	36.0454	36.1119	33.5464	21.3183
FG-O CNT	1	10.9949	16.0716	15.0774	13.4469	14.6154	4.3402
	2	16.1348	22.0695	18.8182	19.0449	14.7550	7.9636
	3	19.3738	31.0727	19.3738	25.5485	16.5016	17.0273
	4	19.3788	32.8351	28.0759	29.7007	19.1524	17.3247
	5	26.6463	34.9777	30.5620	29.7414	22.9926	19.1600
	6	28.2949	38.6394	33.0506	33.0550	30.1205	19.2511
FG-X CNT	1	15.1552	18.9525	18.0228	16.7777	17.7014	6.6028
	2	19.3040	24.5186	19.5714	21.7384	17.7463	9.5252
	3	19.5714	35.0117	21.3967	25.8180	19.1656	18.7035
	4	19.5764	36.3490	30.4141	32.0371	19.3693	19.3737
	5	29.1832	39.0379	35.8724	35.5379	25.3411	22.2820
	6	34.7403	39.6836	37.8550	38.2271	35.6335	23.7887

**Table 8:** The dimensionless first six natural frequencies $\bar{\omega }$ for FG-CNT-reinforced plate with several types of boundary conditions $\left(V_{\mathrm{CNT}}^{*}=0.14,\;a/h=10\right)$.

CNT Distribution	Mode	SSSS	CCCC	CCSS	CSCS	CCFF	CFCF
UD CNT	1	14.6682	18.8542	17.9441	16.5233	17.6226	6.2616
	2	18.8705	24.3743	19.7690	21.4698	17.6727	9.2459
	3	19.7693	34.8746	21.2538	26.0654	19.0664	18.4145
	4	19.7746	36.2671	30.1760	31.7742	19.5794	19.5556
	5	28.7844	39.3845	35.8002	35.3843	25.1267	21.7948
	6	34.4929	39.5926	37.7684	38.0703	35.5519	23.3518
FG-V CNT	1	13.4159	18.4059	17.4633	15.7609	17.1005	5.4974
	2	18.0906	24.1135	19.8706	21.0094	17.1744	8.8533
	3	19.8712	34.7921	20.9318	26.1926	18.6808	18.3042
	4	19.8761	35.5532	30.0544	31.5929	19.6521	19.6276
	5	28.4493	39.0533	35.0697	34.4452	24.9393	20.4615
	6	33.2846	39.5746	37.1960	37.3341	34.7677	22.2670
FG-O CNT	1	11.7336	16.7149	15.7447	14.1157	15.3166	4.7012
	2	16.6636	22.6163	19.3724	19.5683	15.4370	8.1901
	3	19.7233	32.2978	19.7233	26.0009	17.0947	17.4986
	4	19.7283	33.3984	28.5693	30.2258	19.5228	18.0877
	5	27.0960	36.0888	31.7979	31.0146	23.4731	19.5010
	6	29.6777	39.2902	34.1720	34.2161	31.3969	20.1111
FG-X CNT	1	15.8603	19.4936	18.5402	17.3716	18.2150	7.0295
	2	19.9936	25.1816	19.9936	22.4091	18.2580	9.9358
	3	19.9987	35.8970	21.9948	26.3643	19.7081	19.2714
	4	20.0198	37.3880	31.2273	32.9064	19.8096	19.7841
	5	30.0505	39.8323	36.8998	36.5737	26.0403	23.2464
	6	35.7881	40.7784	38.9121	39.3039	36.6646	24.7286

**Table 9:** The dimensionless first six natural frequencies $\bar{\omega }$ for FG-CNT-reinforced plate with several types of boundary conditions $\left(V_{\mathrm{CNT}}^{*}=0.17,\;a/h=10\right)$.

CNT Distribution	Mode	SSSS	CCCC	CCSS	CSCS	CCFF	CFCF
UD CNT	1	17.2282	22.7953	21.6602	19.7729	21.2427	7.2029
	2	22.6414	29.6791	24.3013	26.0099	21.3182	11.0742
	3	24.3016	42.6665	25.8122	32.0048	23.0929	22.4866
	4	24.3082	43.8786	36.8752	38.8103	24.0325	23.9779
	5	35.0543	48.0664	43.2954	42.6982	30.6427	25.8591
	6	41.4851	48.3439	45.8044	46.1123	42.9599	27.9148
FG-V CNT	1	15.5951	22.1523	20.9643	18.7382	20.4814	6.2639
	2	21.6792	29.3329	24.5003	25.4208	20.5947	10.6346
	3	24.5009	42.6051	25.3672	32.2575	22.5474	22.4044
	4	24.5077	42.9122	36.7474	38.6306	24.1940	24.0052
	5	34.7015	47.3646	42.3047	41.3726	30.4090	24.2769
	6	39.7413	48.7211	45.0484	45.0965	41.8897	26.5494
FG-O CNT	1	13.5986	20.0823	18.8814	16.7451	18.3266	5.3426
	2	19.8862	27.4023	23.4006	23.5759	18.4958	9.7936
	3	24.2623	38.9769	24.2623	31.9471	20.5956	21.1536
	4	24.2685	40.6865	34.7258	36.7391	23.9782	21.4110
	5	32.8334	43.7158	38.3598	37.2540	28.4740	23.9263
	6	35.4341	48.2629	41.3522	41.2969	37.8326	23.9562
FG-X CNT	1	18.7939	23.6698	22.4562	20.8923	22.0245	8.1719
	2	24.2372	30.8645	24.6906	27.3472	22.0930	12.0063
	3	24.6906	44.2396	26.8771	32.5214	23.9830	23.7798
	4	24.6969	45.3645	38.4825	40.5259	24.4268	24.3702
	5	36.9439	49.1202	44.7435	44.2607	32.0150	27.6660
	6	43.1742	49.7057	47.3649	47.7933	44.4161	29.6741

**Table 10:** Dimensionless first six natural frequencies $\bar{\omega }$ for an FG-CNT-reinforced plate with simply supported boundary conditions and concentrated mass at the centre $\left(V_{\mathrm{CNT}}^{*}=0.11,\;a/h=10\right)$.

CNT Distribution	$\bar{M}$	First Six Minimum Frequencies					
		1	2	3	4	5	6
UD CNT	0	13.8852	18.1994	19.4225	19.4275	28.1212	33.2913
	0.5	6.3132	18.1999	18.8956	19.4223	19.4272	32.0719
	1	4.5988	18.1999	18.4937	19.4223	19.4272	31.8782
	2	3.2991	18.1999	18.3010	19.4223	19.4272	31.7861
	0	12.6013	17.4092	19.4794	19.4848	27.7626	31.9032
FG-V CNT	0.5	5.9584	17.4093	18.1022	19.4794	19.4844	31.7275
	1	4.3636	17.4093	17.6274	19.4794	19.4844	31.5303
	2	3.1390	17.3983	17.4093	19.4794	19.4844	31.4366
	0	10.9949	16.1348	19.3738	19.3788	26.6463	28.2949
FG-O CNT	0.5	5.3902	16.1348	16.8670	19.3738	19.3788	28.2949
	1	3.9718	16.1348	16.3302	19.3738	19.3788	28.2949
	2	2.8665	16.0686	16.1348	19.3738	19.3788	28.2949
	0	15.1552	19.304	19.5714	19.5764	29.1832	34.7403
FG-X CNT	0.5	6.6718	19.3040	19.5714	19.5764	19.8985	33.0660
	1	4.8415	19.3040	19.5383	19.5714	19.5764	32.8820
	2	3.4667	19.3040	19.3664	19.5714	19.5764	32.7943

**Table 11:** Central transverse deflection of an FG-CNT-reinforced square plate subjected to sin-sin loading $V_{\mathrm{CNT}}^{*}=0.11$.

CNT Distribution	*a/h*	SSSS	CCCC	CCSS	CSCS	CCFF	CFCF
UD CNT	5	0.01216	0.00880	0.01008	0.01040	0.01008	0.03264
	10	0.00502	0.00315	0.00328	0.00397	0.00323	0.01749
	20	0.00300	0.00126	0.00125	0.00182	0.00124	0.01361
	50	0.00241	0.00064	0.00064	0.00115	0.00064	0.01245
	100	0.00232	0.00055	0.00055	0.00105	0.00055	0.01228
FG-V CNT	5	0.01216	0.00912	0.01040	0.01072	0.01008	0.03872
	10	0.00525	0.00336	0.00353	0.00426	0.00349	0.02270
	20	0.00326	0.00153	0.00153	0.00223	0.00152	0.01887
	50	0.00267	0.00094	0.00093	0.00159	0.00093	0.01776
	100	0.00259	0.00085	0.00084	0.00150	0.00084	0.01759
FG-O CNT	5	0.01584	0.01040	0.01216	0.01280	0.01216	0.05216
	10	0.00800	0.00416	0.00443	0.00559	0.00435	0.03142
	20	0.00584	0.00204	0.00207	0.00320	0.00204	0.02670
	50	0.00520	0.00136	0.00135	0.00245	0.00135	0.02536
	100	0.00511	0.00125	0.00125	0.00234	0.00124	0.02516
FG-X CNT	5	0.01104	0.00848	0.00976	0.00976	0.00976	0.02816
	10	0.00420	0.00292	0.00302	0.00353	0.00298	0.01373
	20	0.00219	0.00105	0.00105	0.00145	0.00105	0.00981
	50	0.00160	0.00045	0.00045	0.00078	0.00045	0.00860
	100	0.00151	0.00036	0.00036	0.00068	0.00036	0.00842

**Table 12:** Central transverse deflection of an FG-CNT-reinforced square plate subjected to sin-sin loading $V_{\mathrm{CNT}}^{*}=0.14$.

CNT Distribution	*a/h*	SSSS	CCCC	CCSS	CSCS	CCFF	CFCF
UD CNT	5	0.01136	0.00848	0.00976	0.01008	0.00944	0.02960
	10	0.00447	0.00294	0.00305	0.00363	0.00300	0.01518
	20	0.00251	0.00112	0.00111	0.00159	0.00111	0.01140
	50	0.00194	0.00053	0.00053	0.00093	0.00052	0.01026
	100	0.00186	0.00044	0.00044	0.00084	0.00044	0.01009
FG-V CNT	5	0.01136	0.00848	0.00976	0.01008	0.00976	0.03392
	10	0.00464	0.00311	0.00326	0.00386	0.00319	0.01936
	20	0.00272	0.00134	0.00134	0.00191	0.00133	0.01570
	50	0.00216	0.00076	0.00076	0.00129	0.00076	0.01463
	100	0.00207	0.00068	0.00067	0.00120	0.00067	0.01447
FG-O CNT	5	0.01440	0.00976	0.01136	0.01216	0.01136	0.04544
	10	0.00699	0.00380	0.00403	0.00502	0.00395	0.02640
	20	0.00487	0.00177	0.00178	0.00273	0.00176	0.02208
	50	0.00071	0.00111	0.00110	0.00200	0.00110	0.02084
	100	0.00417	0.00101	0.00100	0.00190	0.00100	0.02065
FG-X CNT	5	0.01040	0.00800	0.00944	0.00944	0.00912	0.02592
	10	0.00382	0.00273	0.00284	0.00328	0.00279	0.01210
	20	0.00186	0.00096	0.00095	0.00129	0.00095	0.00824
	50	0.00128	0.00038	0.00037	0.00064	0.00037	0.00705
	100	0.00120	0.00029	0.00029	0.00054	0.00029	0.00686

**Table 13:** Central transverse deflection of an FG-CNT-reinforced square plate subjected to sin-sin loading $V_{\mathrm{CNT}}^{*}=0.17$.

CNT Distribution	*a/h*	SSSS	CCCC	CCSS	CSCS	CCFF	CFCF
UD CNT	5	0.00768	0.00576	0.00640	0.00672	0.00640	0.02080
	10	0.00321	0.00200	0.00208	0.00252	0.00206	0.01130
	20	0.00195	0.00080	0.00080	0.00118	0.00080	0.00884
	50	0.00158	0.00042	0.00042	0.00075	0.00042	0.00811
	100	0.00152	0.00036	0.00036	0.00069	0.00036	0.00800
FG-V CNT	5	0.00768	0.00576	0.00640	0.00672	0.00640	0.02480
	10	0.00336	0.00214	0.00225	0.00271	0.00221	0.01476
	20	0.00212	0.00098	0.00099	0.00144	0.00098	0.01235
	50	0.00175	0.00061	0.00061	0.00105	0.00061	0.01165
	100	0.00170	0.00056	0.00056	0.00099	0.00055	0.01154
FG-O CNT	5	0.01008	0.00672	0.00768	0.00800	0.00768	0.03328
	10	0.00517	0.00263	0.00279	0.00355	0.00275	0.02054
	20	0.00384	0.00132	0.00133	0.00208	0.00132	0.01763
	50	0.00345	0.00089	0.00089	0.00162	0.00089	0.01681
	100	0.00339	0.00083	0.00083	0.00155	0.00082	0.01669
FG-X CNT	5	0.00704	0.00544	0.00608	0.00640	0.00608	0.01808
	10	0.00269	0.00185	0.00193	0.00225	0.00191	0.00876
	20	0.00142	0.00068	0.00067	0.00094	0.00067	0.00628
	50	0.00104	0.00029	0.00029	0.00051	0.00029	0.00553
	100	0.00098	0.00024	0.00023	0.00045	0.00023	0.00541
